# Introgression among subgroups is an important driving force for genetic improvement and evolution of the Asian cultivated rice *Oryza sativa* L.

**DOI:** 10.3389/fpls.2025.1535880

**Published:** 2025-02-20

**Authors:** Jiawu Zhou, Jing Li, Yu Zhang, Ying Yang, Yonggang Lv, Qiuhong Pu, Xianneng Deng, Dayun Tao

**Affiliations:** Yunnan Seed Laboratory/Yunnan Key Laboratory for Rice Genetic Improvement, Food Crops Research Institute, Yunnan Academy of Agricultural Sciences (YAAS), Kunming, China

**Keywords:** rice, genetic divergence, introgression, genetic improvement, evolution

## Abstract

Anagenesis accumulates favorable mutations that enable crops to adapt to continually improving artificial production environments, while cladogenesis results in the deposition of beneficial variations across diverse ecotypes. Integrating advantageous genetic variations from diverse evolutionary sources establishes the foundation for the continued genetic improvement of crops. For a long time, rice breeding practices have been guided by the established belief that the Asian cultivated rice consists of two subspecies: *Oryza sativa* subsp. *indica* and subsp. *japonica*. Integrating elite genetic variants from both subspecies has been a major strategy for genetic improvement. This approach has proven successful through the achievements of *temperate japonica* breeding programs in China, Japan, and Korea over the past decades. The genetic differentiation within the Asian cultivated rice has been successfully harnessed for heterosis breeding, thereby enhancing rice yield productivity. Genomic investigations have revealed more genetic divergences in the Asian cultivated rice, prompting the proposal of six subgroups within it. This indicates that there is greater potential for uncovering additional genetic divergences and diversity in future breeding practices. Genetic introgression and gene flow among subgroups have led to improvements in agronomic traits within the *indica*, *temperate japonica*, and *tropical japonica* subgroups during the modern rice breeding process. The introgression process has widened the genetic diversity within subgroups and reduced the genetic distance between them, resulting in the creation of new genetic blocks and subpopulations. Artificial introgression has accelerated the evolution process in rice breeding history. Advancements in the study of genetic divergence and diversity in rice offer valuable insights to guide breeding practices. The mini subgroups *aus*, *basmatic*, and *rayada* possess untapped genetic potential but have been poorly studied worldwide; more samples should be further investigated. This information will be invaluable for harnessing these advantageous variations through introgression breeding. Further studying the nature of reproductive barriers among subgroups will enhance our understanding of genetic differentiation, allow us to overcome these barriers and facilitate effective genetic exchange, and even enable us to harness heterosis among subgroups.

## 1 Introduction

Genetic diversity is not only essential for crop resilience in diverse environments but also fundamental to genetic improvement. Enhancing the genetic diversity of breeding pools that carry optimum combinations of favorable alleles for targeted crop-growing regions is crucial for sustaining genetic gain (Bohra et al., 2022). The evolutionary process of crops typically manifests as dynamic changes in genetic diversity, driven by major forces such as migration, selection, genetic drift, reproductive barriers, and polyploidization (Ladizinsky, 1998; Wu et al., 2020; Cao et al., 2023). Migration is the movement of genes within and between populations. When immigrants from other populations bring unique genes, they can increase the genetic variability of the recipient population. When a plant species is domesticated and becomes a crop to sustain human populations, long-term accumulation of genetic diversity occurs through genetic mutation and migration, leading to diffusion and regional expansion of the crop (Ladizinsky, 1998; Hancock, 2012). Genetic improvement is a co-evolutionary process between human civilization and crop species, where new genetic blocks with beneficial characteristics are continuously selected to meet the evolving demands for improved cultivation conditions and higher yields, in order to support the growing human population. Artificial hybridization and selection allow favorable genetic variants dispersed across different populations to be combined into new genetic combinations, and this introgression process accelerates crop evolution. In Asian cultivated rice (*Oryza sativa* L.), the recognition that it consists of two subspecies or groups (Kato et al., 1928) has long been used to guide rice breeding practices aimed at exploiting beneficial genetic diversity and heterosis. The success of breeding programs aimed at genetic improvement of *temperate japonica* rice in Northeast China since the 1950s (Yang et al., 1962) has been demonstrated through inter-subspecific introgression, resulting in significant increases in *indica*-allele frequencies in the cultivars bred after 1990 (Sun et al., 2012). *indica*-*japonica* heterosis, which was expected to show great potential, has been supported by various studies (Yuan, 1987; Fu et al., 2014; Birchler, 2015; Zhang, 2020; Ouyang et al., 2022; Wang et al., 2022a). Advances in genomics have provided deeper insights into the genetic divergence and diversity within the Asian cultivated rice (Garris et al., 2005; McNally et al., 2009; Huang et al., 2012; Xie et al., 2015; Wang et al., 2018; Lin et al., 2020; Qin et al., 2021; Wang et al., 2022b; Ye et al., 2022), highlighting the role of introgression among subgroups and offering a more comprehensive understanding of the genetic makeup and evolutionary history of this crop.

However, the progress of human society and population growth are exerting increasing pressure on rice production and productivity. Therefore, accurately and effectively utilizing the dispersed and rich variations in the Asian cultivated rice to develop new varieties that can address constantly emerging problems in rice production, such as climate change, water scarcity, overuse of fertilizers, insecticides, antibiotics, and herbicides, and thus reduce the cost of rice production and environmental pollution, is a fundamental issue.

Therefore, a scenario for rice genetic improvement that involves integrating elite genetic variations into new combination blocks through inter-subgroup introgression was outlined. Additionally, how the genetic structure within and between subgroups of Asian cultivated rice has evolved and changed over time, highlighting key genetic variations and patterns that have emerged, was reviewed, too. Then, that further emphasis should be placed on mining favorable alleles in previously rarely mentioned subgroups, including *aus*, *basmatic*, and *rayada* (Bin Rahman and Zhang, 2013; Wang et al., 2013; Jing et al., 2023), since these subgroups contain favorable traits that have not been fully identified and utilized worldwide, such as adaptation to water-insufficient upland conditions, robust growth in low-fertilizer environments, and strong competition with weeds, which could be exploited for genetic improvement of other subgroups. Furthermore, it is crucial to conduct investigations into whether hybrid vigor exists among six subgroups of the Asian cultivated rice. Additionally, examining the nature of hybrid sterility between these subgroups will be essential for the future utilization of heterosis among them.

## 2 The Asian cultivated rice continuously accumulated favorable genetic variations during anagenesis

The Asian cultivated rice, *O. sativa*, was domesticated from the wild species, *Oryza rufipogon* and *O. nivara*. During the domestication process, a series of obvious changes occurred in morphological traits, physiological characteristics, and ecological adaptability. These changes include the transition from creeping to erect growth, the loss of grain shattering, the shortening or absence of awns, changes in hull and grain color, reduced grain dormancy, alterations in panicle architecture, increased grain number and weight, and improved regional adaptability. These changes have collectively contributed to the improvement of agronomic traits that were favored by our human ancestors (Xu and Sun, 2021). One typical example is the amylose content of rice grain, which is an important factor in determining the eating quality of rice. The diverse amylose content in rice is mainly attributed to mutations and selections in the *Waxy* gene, transitioning from the ancestral allele *Wx^lv^* in wild ancestors to the *indica*-type allele *Wx^a^*, the *temperate japonica*-type allele *Wx^b^*, the *tropical japonica*-type allele *Wx^in^*, and the intermediate-type allele *Wx^op^*. Furthermore, the *Wx^mp^* and *wx* alleles were derived from mutations of the *Wx^b^* allele (Zhang et al., 2019). For the last century, modern rice breeding activities have endowed the Asian cultivated rice with more trait improvements. For example, modern rice cultivars with a semi-dwarf plant type that adapts to higher fertilizer inputs and increased planting density without lodging have been bred. These cultivars provide more food to feed the growing population of the world. These improvements are accompanied by a series of favorable mutations and allelic variant accumulations in the crop. Consequently, some favorable alleles, such as various dwarf alleles of *Sd1*, have become prevalent in modern rice cultivars (Chen et al., 2020; Sha et al., 2022).

The evolution of *O. sativa* involves the fixation of favorable variants/alleles and the elimination of inferior ones under cultivation conditions, reflecting the dynamic genetic diversity throughout its evolution. Over time, original unique alleles are lost, while new unique alleles are added (Yonemaru et al., 2012). Over decades of breeding, the total number of alleles, the number of unique alleles, and the number of varieties with unique alleles tend to increase in *O. sativa* (Tang et al., 2021). When compared to *O. rufipogon*, the diversity of the entire *O. sativa* population has been reduced by only ~10% of its ancestors (Huang et al., 2012; Wang et al., 2022b).

## 3 Cladogenesis resulted in genetic diversification among subgroups of the Asian cultivated rice

**Figure 1:**
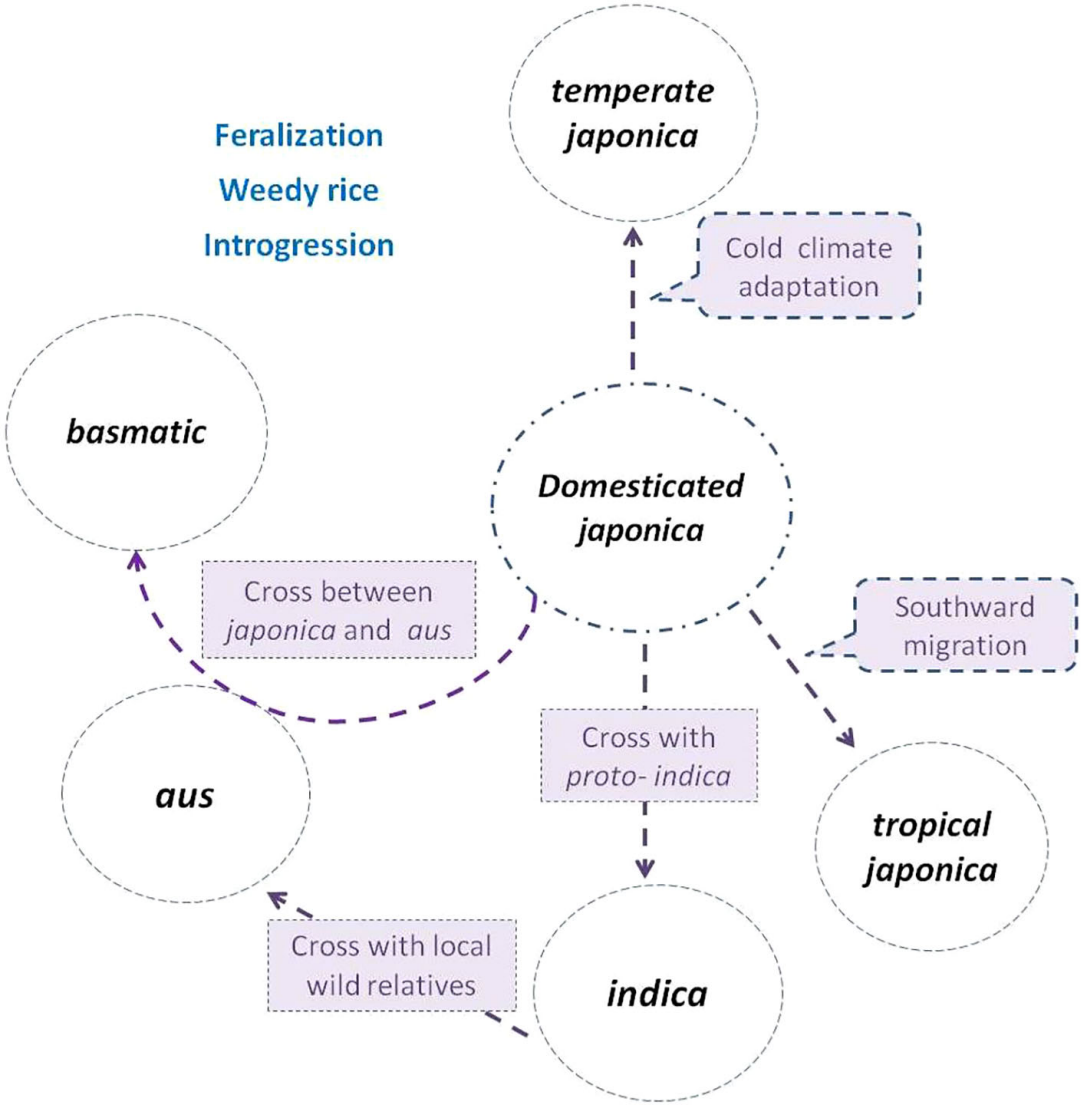
Subgroup differentiation of *O. sativa* in the evolution process (Zhou et al., 2022) *japonica* rice (circled in the center) was first domesticated from *Oryza rufipogon*, then diversified northward as *temperate japonica* and southward as *tropical japonica* (Gutaker et al., 2020). *indica* descended from hybridization between *japonica* and local wild populations or proto-*indica* (Fuller, 2011; Gross and Zhao, 2014). *aus* was derived from the hybridization between *indica* and local wild populations, while *basmatic* was derived from hybridization between *japonica* and *aus* (Civán et al., 2015; Kim et al., 2016; Wang et al., 2017; Choi et al., 2020). Weedy rice strains de-domesticated from and coexisted with cultivated subgroups, and frequently crossed with wild populations (if present) or landraces during their evolutionary process (Li and Olsen, 2020; Vigueira et al., 2020).

As one of the most important crops in the world, rice (*O. sativa* L.) is widely distributed in a range of tropical, subtropical, and temperate climates, from 55°N in China to 36°S in Chile. This far exceeds the distribution range of its ancestors, *O. rufipogon* and *O. nivara*, which are primarily limited to swampy habitats in humid tropical Asia. It evolved various ecotypes in different ecosystems, such as irrigated, lowland, upland, and flood-prone areas, and expanded its range to high latitude and altitude temperate climate conditions. Additionally, it accumulated numerous favorable variants, for instance, glutinous rice harboring the *wx* haplotype, which was absent in its wild progenitors (Chatterjee, 1948; Morishima et al., 1961; Morishima, 1969; Khush, 1997). It was estimated that more than 4,120,000 rice cultivars and germplasm accessions have been recognized worldwide (Song et al., 2021). For a long time, it has been widely accepted and recognized that cultivars of *O. sativa* are classified into two major types: *O. sativa* subsp. *indica* and subsp. *japonica*, based on morphological, serological characters, as well as inter-varietal hybrid fertility (Kato et al., 1928). Based on morphological characters, some researches proposed that it could be grouped in three types, A, B, and C (Matsuo, 1952), or *indica*, *javanica*, and *japonica* (Morinaga, 1954), or *indica*, *tropical japonica*, and *temperate japonica* (Oka, 1958). One thousand six hundred and eighty-eight traditional rice varieties of the Asian cultivated rice were clustered into six groups based on isozyme polymorphic markers (Glaszmann, 1987), which roughly coincided with different ecological types and geographical distributions. Subsequent researchers proposed that the Asian cultivated rice can be classified into five subgroups: *indica*, *aus*, *aromatic*, *temperate japonica*, and *tropical japonica*, using SSR markers (Garris et al., 2005), SNPs, and genomic sequence data (McNally et al., 2009; Wang et al., 2018; Chen et al., 2019). Since the term ‘*aromatic*’ implies fragrant rice, it is not appropriate to use it to refer to a group of rice that includes both fragrant and non-fragrant varieties, which are specifically found in South Asia and its surrounding regions. Therefore, we refer to this group as “*basmatic*” instead of “*aromatic*” (Zhang et al., 2022; Zhou et al., 2022) (**Figure 1**). A recent investigation suggested that the Asian cultivated rice consists of two separate monophyletic groups, representing the two subspecies. The subspecies *indica* encompasses two subgroups: *indica* and *aus*. Meanwhile, the subspecies *japonica* includes four subgroups: *aromatic* (also known as *basmatic*), *rayada*, *temperate japonica*, and *tropical japonica*. Notably, three minor subgroups—*aus*, *basmatic*, and *rayada*—are genetically distinct, suggesting that they deserve further attention despite being cultivated in limited areas in South and Southeast Asia (Jing et al., 2023). Furthermore, some investigations indicated that both *aus* and *rayada* exhibit high levels of genetic diversity and genetic differentiation (Parsons et al., 1999; Cai and Morishima, 2000; Wang et al., 2013; Travis et al., 2015; Islam et al., 2018). While Civán et al. (2015) proposed that the origin of *aus* was parallel to that of *indica* and *japonica* rice.

Generally, subgroup or subpopulation differentiation is a common phenomenon in the Asian cultivated rice, adapting to various ecological conditions at both the species level and the subspecies levels of *indica* and *japonica*. Greater genetic divergence and diversity implies further potential for unlocking available genetic variation in future breeding practices.

## 4 Introgression among subgroups is the mainstream approach for genetic improvement and an important driving force in the evolution of Asian cultivated rice

Artificial hybridization in crop plants, which began about 200 years ago, has allowed breeders to compile traits from various landraces and cultivars into a single plant (Ladizinsky, 1998). For example, the *NRT1.1b* gene, conferring nitrogen utilization efficiency (NUE), carries a beneficial allele in *indica* varieties. The *DEP1* gene, which influences panicle architecture and yield, has a favorable allele in *japonica* varieties. When the *nrt1.1b* allele was incorporated into *japonica* lines carrying *dep1*, there was a significant increase in nitrate reductase activity, nitrogen uptake, and grain yield under low nitrogen conditions. Conversely, introducing the *dep1* allele into *indica* lines already carrying the *nrt1.1b* allele resulted in enhanced glutamine synthetase activity, improved nitrogen transfer, and increased grain yield under both low and high nitrogen conditions, ultimately boosting yield potential in specific planting environments (Zhao et al., 2017).

### 4.1 *indica-temperate japonica* introgression for *temperate japonica* rice improvement

*indica-temperate japonica* introgression has played an important role in *temperate japonica* breeding practices in Northeast China, Korea and Japan. In the early breeding stage of *temperate japonica* rice, trait improvement was primarily achieved through hybridization between *temperate japonica* varieties and subsequent artificial selection, which aimed to pyramid and fix superior alleles of key genes. However, this process resulted in a narrowing of the genetic background, compared to the prior stage (Cui et al., 2022). To broaden the genetic basis of *temperate japonica* cultivars, hybridization and introgression between *temperate japonica* and *indica* varieties were practiced in Northeast China in the 1950s (Yang et al., 1962). Favorable traits from *indica* rice genome were integrated into *temperate japonica* background through this method. Most of the prevalent commercial rice varieties grown in Northern China since the 1980s were bred from crosses between *indica* and *temperate japonica* (Jiang et al., 2022). The frequency of *indica* alleles was significantly increased in cultivars bred after 1990. These alleles were positively and significantly correlated with the number of spikelets per panicle, and negatively and significantly correlated with the number of panicles per plant. Specifically, favorable *indica* alleles of *GN1a* and *GS3* were partially fixed in the genome of northern *temperate japonica* cultivars. In contrast, inferior *indica* alleles of *Wx* and *qSH1* were eliminated during the breeding process, while favorable *temperate japonica* alleles of *DEP1* and *qSW5* were retained (Sun et al., 2012). A large introgression segment from *indica*, located on chromosome 12, was detected in 24% of the *temperate japonica* samples. This segment covers many functional genes, including two rice blast resistance genes, *Pi-ta* and *Ptr*. Another high-frequency introgression from *indica*, also on chromosome 12, was present in approximately 19% of the *temperate japonica* samples. This introgression harbors a QTL, *qS12*, associated with hybrid male sterility. On chromosome 11, introgressions from *indica* were detected in 12.1% of the *temperate japonica* samples, which contain a gene related to rice stripe virus resistance, *STV11* (Chen et al., 2020). Favorable alleles of *IPA1, SMG1, Dep3, Ghd7, GW5, OsPIN3t, xa13, Bph3, Pia, Pib*, and *Pi-d2* were introduced from *indica* rice into *temperate japonica* rice, This facilitated the recombination of the gene pools of *temperate japonica* and *indica*, resulting in the creation of new recombinant blocks and the improvement of agronomic traits in *temperate japonica* cultivars. Consequently, this contributed to the modern breeding of improved plant types, transitioning from multi-tiller plants to moderate-tiller plants with larger panicle sizes and enhanced blast resistance (Cui et al., 2022). Over the past fifty years, the *indica*-*temperate japonica* hybridization breeding process has achieved significant genetic gains in *temperate japonica* varieties in Northeast China. During this period, *indica* introgressions were detected on all 12 chromosomes. The introgression of *indica* segments carrying *qSB2*, *qSB8*, and *qSB10* led to a 100% increase in secondary branching number per panicle and a 50% increase in grain number per panicle. In Heilongjiang Province, Northeast China, the average content of *indica* introgressions in a cultivar has increased markedly over time, from 8.2 Mb before 1980 to 76.6 Mb after 2010. Notably, the two adjacent rice blast resistance loci, *Pi-ta* and *Ptr*, which originate from *indica* and have beneficial effects, have significantly enhanced blast resistance in *temperate japonica* rice (Chen et al., 2023). The last century has witnessed tremendous leaps in China’s rice productivity, from 1.9 t/ha in 1949 to 7.0 t/ha in 2018 (http://faostat.fao.org/). This significant increase in rice productivity can be primarily attributed to the revolution of semi-dwarf and hybrid rice varieties. Additionally, the rapid expansion of high-yielding *temperate japonica* varieties that incorporate *indica* introgressions has also played a partial role (Wang et al., 2023a). In the national new rice varietal trials conducted in Northeast China, the average yield of new *temperate japonica* varieties increased from 8.3 t/ha in 2004 to 9.0 t/ha in 2018 (Fei et al., 2020). In South China, particularly in Jiangsu Province, 278 *temperate japonica* rice varieties with low amylose content, disease resistance, high yield, and excellent eating quality were released between 2001 and 2021. These varieties integrated favorable alleles from both *japonica* (*Wx^mp^*, *Badh2*) and *indica* (*Gs3*, *Stv-bi*, *Pi-ta*, *Pi-b*) sources. The breeding of *temperate japonica* rice with low amylose content, high yield, and multiple disease resistances has become an important direction for enhancing the taste and quality of rice in the middle and lower reaches of the Yangtze River (Wang, 2022c).

### 4.2 *japonica-indica* introgression for *indica* rice improvement

In India, *indica-japonica* introgression breeding program initiated in 1952 (Chakraborti et al., 2021), The International Rice Commission (IRC) was focusing to cross tall “*indica*” rice to dwarf “*japonica*” rice to evolve with shorter rice varieties, and few varieties developed by IRC made greater impact on rice growing farmers, One *indica* × *japonica* breeding line Mahsuri became popular in different countries (Patra et al., 2020).

The Tongil rice, developed in 1972, emerged as a high-yield rice variety that played a pivotal role in achieving staple food self-sufficiency in Korea during the renowned ‘Korean Green Revolution’. This groundbreaking cultivar represented the first successful example of *japonica*-*indica* hybridization in Korea. An in-depth analysis of its genome structure reveals that the Tongil genome is predominantly composed of the *indica* genome, with a modest yet significant proportion of *japonica* genome introgression (Kim et al., 2014). In Japan, high-yielding rice cultivars have been successfully developed through crosses between overseas *indica* varieties and domestic *japonica* varieties. Among these cultivars, some exhibit *indica*-type characteristics (Yonemaru et al., 2014). Minghui 63, a famous *indica* restorer parent of three-line hybrid rice from China, shows an introgression on chromosome 6 (0–4 Mb) from *japonica.* This introgression coincides with QTLs reported in several studies to be associated with various traits, including leaf area, vascular bundle number, root features, plant height, cooking quality, and amylose content (McNally et al., 2009). All of the *indica* varieties carrying the introgression of *temperate japonica Wx^b^* allele exhibit significantly lower amylose content (Zhao et al., 2010). About 63% of the *indica* samples carried introgression from *japonica* that introduced the *Wx^b^* allele, leading to a reduction in amylose content in rice grains and subsequently improving eating quality. The *japonica* introgression in 45% of the *indica* samples carried the *japonica* allele of the *ALK* gene, which was reported to reduce grain gelatinization temperature, also contributes to eating quality (Chen et al., 2020).

Breeding practices increased mutual introgression between the two subgroups, likely increasing within-population diversity and reducing subgroup differentiation. Interestingly, there was greater gene flow from *japonica* to *indica* than from *indica* to *japonica*, which was obviously due to the increased inter-subgroup crosses made in many rice breeding programs (Zhang et al., 2021).

### 4.3 Introgression of *tropical japonica* germplasm to develop new plant type *indica*

To break the yield potential barrier, IRRI scientists proposed the high-yielding *indica* new plant type in the late 1980s and early 1990s. The first-generation NPT lines developed at IRRI through hybridization between *indica* and *tropical japonica* had large panicles, few unproductive tillers and resistance to lodging but low grain yield due to the limited production of biomass and poor filling of grains (Khush, 1995). The second-generation NPT lines were obtained by crossing elite *indica* with improved *tropical japonica*, in which, genes from *indica* parents have efficiently decreased panicle size and enhanced tiller ability. *indica* germplasm also helped to improve other NPT characteristics such as quality of grains and disease and insect resistance (Rahangdale et al., 2019). In Indonesia, the NPT rice architecture allows a higher yield than the green revolution rice type represented by the Ciherang variety. The yield advantage is 1.67 t ha^-1^ or 26% based on the best-lines average (Aswidinnoor et al., 2023).

### 4.4 Introgression from *indica/aus* re-shaped the genome of *tropical japonica*

Hybrid introgression among subgroups has renovated the genomes of improved cultivars by integrating favorable alleles from exogenous germplasms during the breeding practices of the past century. There is a highly elevated level of introgression from *indica* into many *tropical japonica* varieties near the *Sd1* gene. *Pi-ta* is known to be of *indica* origin, but because *tropical japonica* varieties are best adapted to upland growing conditions, breeders have frequently introgressed this resistance gene to enhance the productivity of *tropical japonica* cultivars. An extensive segment of *indica* DNA located in the centromeric region on chromosome 12 is found in several *tropical japonica* accessions. The *Pi-ta* gene in the US varieties can be traced back to the cultivar, Tetep, a Vietnamese *indica* accession (McNally et al., 2009; Zhao et al., 2010). The *tropical japonica* accessions that exhibit introgression have identical haplotype with *indica* accessions. The *tropical japonica* population acquired some favorable alleles of phenotype-related loci, such as heat tolerant gene *TT1*, rice quality genes *Wx*, *ALK*, *BAD2*, and grain shape gene *Gs3*, from the *indica* through genetic introgression. Excessive genetic introgression is observed between the *indica* subgroup and the *tropical japonica* subgroup, which may be due to the overlap in their distribution areas, both located in the southern part of Southeast Asia. Thus, *tropical japonica* may have originated from ancient *japonica* through genetic introgression and developed into a unique ecotype after acquiring numerous alleles from *indica* to adapt to the local environment in the southern part of East Asia (Gong and Han, 2022).

Moroberekan, a *tropical japonica* traditional variety from Africa and a popular donor for disease resistance and drought tolerance, contains several regions on chromosome 6 introgressed from *indica* or *aus*, one of which colocalizes to a large cluster of NB-ARC-type resistance genes between 9.2 and 11.1 Mb (McNally et al., 2009).

In 3K project, within Chromosome 1, seven *tropical japonica* accessions from Asia share an *indica/aus* haplotype of more than 10 Mb (from 9.2 Mb to 19.3 Mb). The predominant *japonica* subgroup in Africa is *tropical japonica*. A 3.8 Mb introgression on chromosome 6 from *aus* is shared by 14 *tropical japonica* accessions from West Africa. Additionally, two accessions share a shorter *aus* haplotype between 17.9 and 20.26 Mb. On the end of chromosome 6, between 25.8 and 26.7 Mb, a block of haplotype of *aus* ancestry was found in half of the *tropical japonica* accessions from Africa. The above results suggest that hybridization and introgression between the *tropical japonica* accessions and the *aus* subgroup played a role in the generation of West African upland accessions. Out of a total of 46 genes underlying this introgression, some genes have functions directly related to responses to abiotic stresses such as salt stress and water stress. The presence of ABA receptor genes (*OsPYL/RCAR7, OsPYL/RCAR8*, and *OsPYL7*) from the introgression of *aus* suggests their function in water stress responses. Several studies have highlighted the high adaptability of the *aus* ecotype to drought or heat. African upland rice varieties, exemplified by Moroberekan, are known for their remarkable drought tolerance. An adaptive introgression on chromosome 6 derived from *aus* bears genes potentially involved in drought responses. This work illustrates how the evolution of genetic diversity along geographic migration can be used to enhance the corpus of genes involved in crop adaptation (Beye et al., 2023).

### 4.5 *aus - japonica* introgression shaped *basmatic* genome in early evolution stage

Genomic investigations suggestion that the *basmatic* rice is a result of the hybridization between *japonica* and *aus* in early evolution stage (Civán et al., 2015; Kishor et al., 2020). The *japonica* rice contributed the highest amount of genetic material to *basmatic*, while strong evidence of admixture between the *basmatic* and *aus* groups was detected (Choi et al., 2020). A unique pericarp color haplotype, *rc-s*, is shared only by *aus* and *basmatic* subgroups (Sweeney et al., 2007). The 3K project found that about 65% of the *basmatic* genome was derived from *aus*, while the remaining 35% from *japonica*, suggesting *basmatic* originated from crosses between *aus* and *japonica* (Wang et al., 2022b).

### 4.6 Genomic differentiation and introgression shaped rice heterosis

Historical efforts to genetically improve hybrid parental lines have led to significant increases in commercial hybrid rice productivity in China, from approximately 3.8 t/ha in the 1970s to approximately 6.8 t/ha in recent years (Yuan, 2015). The superior performance of the hybrid rice may have resulted from independent improvements in the two rice subpopulations within *indica* (Xie et al., 2015). Geographic adaptation and the accumulation of divergent selections in distinct breeding pools may cause differentiation within the *indica* subgroup. Genome clustering analysis can divide *indica* into several divergent subpopulations, two of which are consistently aligned with the hypothetical two heterotic groups in the germplasms of *indica* rice: short-statured varieties of South China origin and medium-height lines of Southeast Asia origin. *ind*-3B, corresponding to the maintainer lines of three-line hybrid rice, and *ind*-3R, the restorer lines of three-line hybrid rice, may represent two heterotic groups in Chinese *indica* rice (Xie et al., 2015; Li et al., 2020; Chen et al., 2020).

The genetic differences that cause hybrid vigor between male and female parents in hybrid rice breeding history in China involve exogenous genetic introgression. The male parents of hybrid rice varieties are either introduced directly from the International Rice Research Institute (IRRI) or bred using IRRI varieties as donor parents (Xie and Zhang, 2018). This means most of the restorer lines share more than 40% ancestry from IRRI varieties (Zhao et al., 2022). During hybrid breeding, regions that were divergently selected from subgroups including *indica*, *aus*, and *japonica* were first introgressed into hybrid parents. These introgressed regions were then selected based on their effect. The introgressed regions explained an average of 41.44% of grain yield heritability, 45.46% of biomass heritability, 45.86% of tiller number heritability, and 35.02% of panicle weight heritability in the hybrids (Lin et al., 2020). More than thirty-megabase (33.3-Mb) differentiated regions were identified between the *ind*-3B and *ind*-3R subpopulations. Many of the agronomically important genes, such as *NOG1*, *LAX1*, *SD1*, *Wx*, *RFT1*, *Hd3a*, and *Hd1*, fell within these differentiated regions. The heterotic loci *Hd3a* and *TAC1*, distributed differentially between the female and male parents of three-line *indica* hybrid rice, indicate their potential contribution to heterosis. *Hd3a* was present in over 98% of *ind*-3R samples, while this ratio was less than 30% in *ind*-3B subpopulation. In contrast, the introgression surrounding *TAC1* on chromosome 9, from *japonica*, was present in 67.1% of the *ind*-3B samples, while only 5.1% of the *ind*-3R samples carried this introgression (Chen et al., 2020). Differential introgressions formatted heterotic groups (Chen et al., 2020; Lin et al., 2020). Minghui 63, the famous restorer parent of *indica* three-line hybrid rice, shows an introgression on chromosome 6 (0–4 Mb) from *japonica*, which also contains heterotic loci associated with high yield performance in heterozygous *vs*. homozygous individuals (McNally et al., 2009).

### 4.7 Breeding practices created new haplotype blocks and new subpopulations

Landraces typically exhibit unique characteristics that make them adapt to their local environments, aligning with the interests of local farmers and consumers. However, they often contain linkage drags or detrimental traits. In breeding practice, hybridization and selection of several local varieties or accessions are used to combine favorable traits and eliminate unfavorable ones, resulting in newly combining blocks. Past breeding generally increased haplotype diversity in modern varieties compared with landraces in both *indica* and *japonica* (Zhang et al., 2021).

Most modern Japanese rice varieties were bred from crosses among a limited number of oldest varieties or landraces. However, phylogenetic analysis suggests that a strong population structure developed in Japanese cultivars during modern breeding activities. As a result, the genetic structure of modern varieties significantly differed from those bred before 1922 and landraces. Over the 75 years from 1931 to 2005, the number of new haplotype blocks gradually increased as cultivars underwent modern breeding (Yonemaru et al., 2012).

*indica* is the largest subgroup in *O. sativa*, accounting for over 80% of total rice cultivation worldwide. It is the staple food for most people in tropical and subtropical regions of South China, Southeast Asia, and South Asia (Mahesh et al., 2016). Modern breeding practices have greatly changed the *indica* population structure. Based on genomic sequence data, 809 worldwide *indica* varieties were classified into two groups: IndI and IndII. The IndI group had germplasm mainly of South China origin, while IndII mainly originated from South Asia and Southeast Asia. The differentiation between IndI and IndII might be caused by geographic adaptation and the accumulation of divergent selections in distinct breeding pools (Xie et al., 2015). In the 3K project, *indica* was divided into four clusters: XI-1A from East Asia, XI-1B of modern varieties of diverse origins, XI-2 from South Asia, and XI-3 from Southeast Asia (Wang et al., 2018). Based on the frequency differentiation of local haplotypes from sequencing data of 2,429 *indica* accessions, three groups were classified. One group mainly originated from China, another from South Asia and Southeast Asia, and the third distinct group was mainly constituted of modern varieties with diverse origins. The modern *indica* subgroup exhibits signatures of gathering favorable alleles for rice production (Qin et al., 2021). In a recent investigation, *indica* was divided into two groups: *indica*-I (IND-I) and *indica*-II (IND-II). Most of the Chinese rice varieties belonged to IND-II, which was further classified into two subpopulations. The IND-C1 subpopulation was mainly composed of land cultivars bred in the 1950s, while the IND-C2 subpopulation included modern cultivars bred after the 1980s (Ge et al., 2022). Modern varieties with diverse origins formed a separate subpopulation that strongly represented beneficial alleles related to agricultural production. This genetic differentiation did not occur on a genome-wide scale, but rather was confined to specific loci or chromosome intervals (Xu et al., 2016; Wang et al., 2018; Qin et al., 2021). Genetic divergences in the *indica* group demonstrate parallel evolution in ecosystems. Subsequently, breeding preferences under certain spatial and temporal conditions also impact the result of classification. This may be due to certain variety groups harboring similar allelic combination blocks by using similar backbone parents or popular alleles, such as *Sd1*, during a specific breeding phase. Conditions and preferences during this phase can also influence allelic combinations. Therefore, long-term regional distribution, geographical barriers, and breeding efforts are the major factors contributing to genetic divergence in the *indica* subgroup.

**Figure 2:**
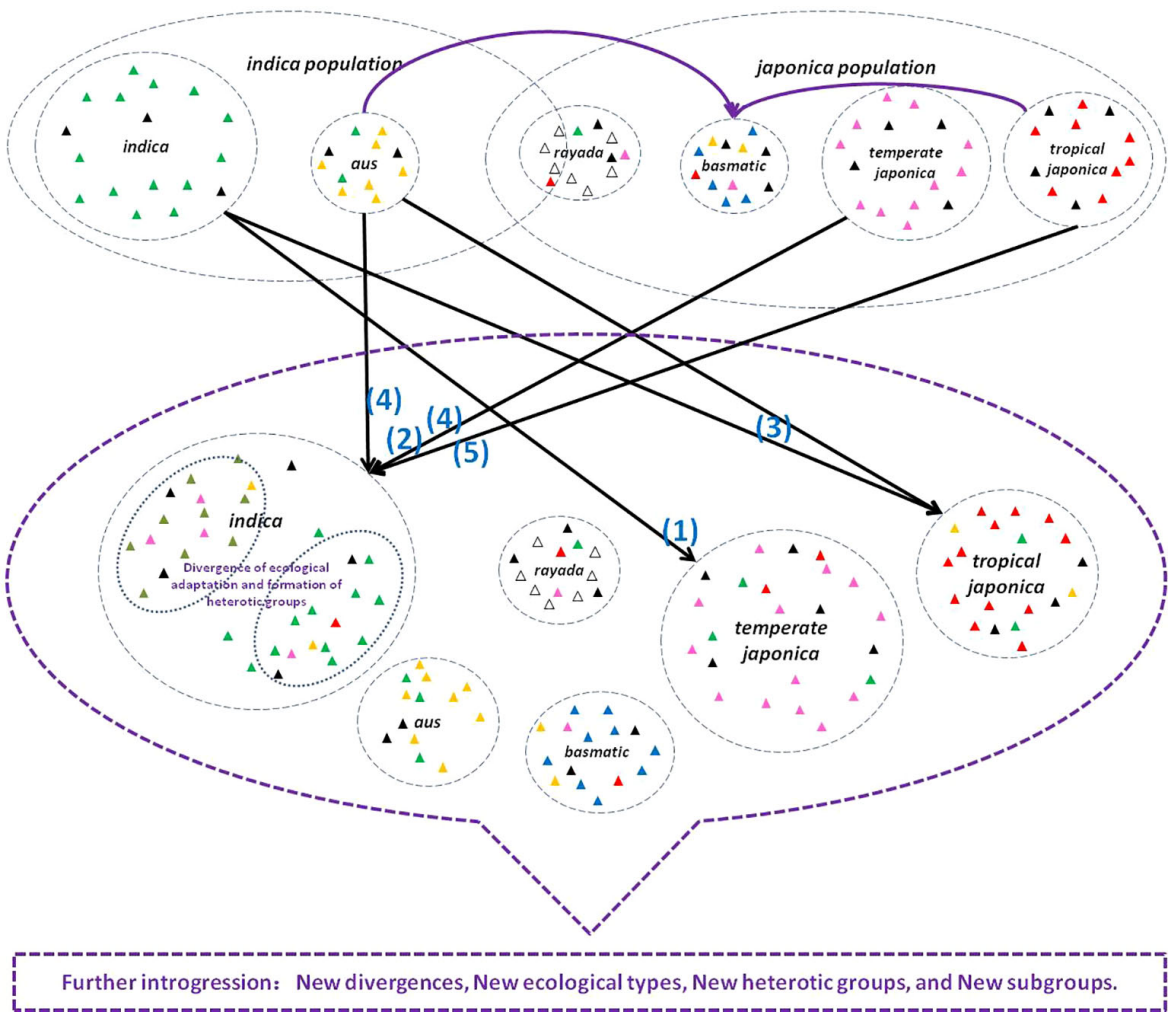
Introgression impacts genetic divergence, diversity in the evolution process of *O. sativa*. Triangles of various colors represent special variants or mutations that have occurred during the evolutionary process. Purple solid arrows denote the direction of introgression during the domestication process and early evolution stages. These introgressions played a role in the formation of the *basmatic* genome by integrating genetic material from *japonica* and *aus* (Civán et al., 2015; Choi et al., 2020). Black solid arrows indicate introgression and gene flow during the modern breeding process, (1) *indica* introgrsssion for *temperate japonica* improvement in northeast China (Sun et al., 2012; Chen et al., 2020; Cui et al., 2022); (2) *japonica* introgression for *indica* improvement in China, India, Japan, and Korea (Zhao et al., 2010; Kim et al., 2014; Yonemaru et al., 2014; Patra et al., 2020; Chakraborti et al., 2021). *indica/aus* intrgression for *tropical japonica* improvement (McNally et al., 2009; Gong and Han, 2022; Beye et al., 2023); (4) *aus/japonica* introgression shaped heterotic group (McNally et al., 2009; Chen et al., 2020; Lin et al., 2020); (5) *tropical japonica* introgression for the development of NPT *indica* (Khush, 1995; Aswidinnoor et al., 2023). The gene flow between subgroups without literature reference is not shown in the figure. The purple dashed circle signifies further introgression among different subgroups.

Generally, Asian cultivated rice has undergone a remarkable evolution in adapting to improved cultivation environments. Variants that were adapted to specific ecosystems were selected, leading to the formation of unique haplotypes. Favorable mutations were fixed and accumulated within these ecosystems, ultimately resulting in the formation of subgroups, subpopulations, or ecotypes (**Figure 1**; Zhou et al., 2022). Mutual introgression among subgroups can create new recombination and genetic blocks. Intensive artificial introgression and selection further enhance genetic migration and genetic drift, which sharply accelerates the evolution process (**Figure 2**).

## 5 Further genomic introgression among subgroups for the changing world

Artificial hybridization in crop plants began about 200 years ago. It has enabled the assembly of traits, which individually exist in several landraces and cultivars, into one plant (Ladizinsky, 1998). In rice, the frequencies of favorable haplotypes are typically very low in modern *indica* and *japonica* varieties for most genes. However, favorable alleles at most cloned genes are present at high frequencies in landraces from specific geographic regions. Unfortunately, accessions from these populations have rarely been used as breeding parents. Specifically, the *tropical japonica*, subtropical *japonica*, and *aus* subgroups have high frequencies of favorable haplotypes at most genes. These haplotypes are present at low frequencies in modern varieties. As a result, most modern varieties currently grown in farmers’ fields do not have the ‘best’ alleles at most gene loci. Therefore, there is a huge potential to improve yield traits and productivity of modern varieties by pyramiding the missing favorable haplotypes at multiple loci from carefully selected breeding parents (Zhang et al., 2021).

*indica*-*japonica* introgression is progressing well in rice breeding practices and has achieved remarkable results. However, there has been limited progress in utilizing favorable variations from other subgroups, especially the mini-subgroups such as *aus*, *basmatic*, and *rayada*, in which distinct characters are worthy of more attention (Bin Rahman and Zhang, 2013; Jing et al., 2023). For instance, the favorable allele for higher nitrogen use efficiency (NUE) found in *aus* and *basmatic* rice can be utilized to enhance this trait in *japonica* and *indica* rice (Liu et al., 2021), thereby alleviating the increasing application of nitrogen fertilizers and corresponding environmental pollution issues. Favorable alleles that affect rice quality, identified in most *basmatic* rice varieties (Siddiq et al., 2012; Babar et al., 2022), could be exploited to develop new favorable genetic combinations in the backgrounds of other subgroups, thereby improving rice quality. The combination of population growth and restrictions on available arable land has intensified genetic improvement efforts and the intensive cultivation of *temperate japonica* rice in East Asia, particularly in Northeast China, Japan, and Korea. This has resulted in compact plant types and superior yield traits among *temperate japonica* varieties in this region (Fei et al., 2020; Cui et al., 2022; Wang et al., 2023a). At the same time, increased fertilizer application and management inputs are required to ensure sufficient biomass and grain production. In comparison, rice varieties from South and Southeast Asia, including *indica*, *aus*, *basmatic*, and *tropical japonica*, can achieve good biomass and exhibit resistance/tolerance to biotic and abiotic stresses even under low-fertilizer and low-input conditions (**Table 1**).

**Table 1:** Some favorable characters/genes/QTLs from different subgroups of *Oryza sativa*.

Subgroups	Traits	Genes/QTL	References
*aus*	P-uptake	*Pup1, Pstol1*	Kokaji and Shimizu, 2022
*aus*	Drought-tolerant	*qDTY1. 1*	Ghimire et al., 2012
*aus*	Heat tolerance	*qHTSF4. 1*	Ye et al., 2015
*aus*	Resilience to yield reductions triggered by climate change		Ara et al., 2017
*aus*	Drought tolerance	*qtl12.1*	Bernier et al., 2009
*aus*	BB resistance	*xa5*	Garris et al., 2003
*aus*	Deep water resilience	*SNORKEL1, SNORKEL2*	Hattori et al., 2009
*aus*	Submergence tolerance	*Sub1A*	Xu et al., 2006
*aus*	Wide-compatability in hybrid sterility locus	*S5n*	Schatz et al., 2014
*aus*	Culm number	*SLR1*	Zhang et al., 2021
*aus*	Culm number	*OsSPL7*	Zhang et al., 2021
*aus*	Culm number	*LGD1*	Zhang et al., 2021
*basmatic*	Fragrance	*BAD1, BAD2*	Bradbury et al., 2005
*indica*	Heat tolerance	*TT1*	Gong and Han, 2022
*indica*	Low-Phosphorus tolerance	*qLPTN6, qLPMRL6*	Kokaji and Shimizu, 2022
*indica*	Improving net leaf photosynthetic rate	*qCAR5, qHP10*	Yamashita et al., 2022
*indica*	Increasing tiller number	*OsAAP3*	Lu et al., 2018
*indica*	Bud elongation	*OsAAP3*	Lu et al., 2018
*indica*	Nitrogen use efficiency	*OsAAP3*	Lu et al., 2018
*indica*	Grain yield	*OsAAP3*	Lu et al., 2018
*indica*	High nitrogen-use efficiency and grain yield	*NRT1.1B*	Hu et al., 2015
*indica*	Blast-resistant	*pi-ta*	Jia et al., 2003
*indica*	Grain number per panicle	*Gn1-a*	Ashikari et al., 2005
*indica*	Grain number per panicle	*NOG1*	Huo et al., 2017
*indica*	Grain number per panicle	*LAX1*	Komatsu et al., 2003
*indica*	Plant architecture	*IPA1*	Miura et al., 2010
*indica*	Grain number per panicle	*NOG1*	Li et al., 2020
*indica*	Grain length	*GLW7*	Li et al., 2020; Gong and Han, 2022
*indica*	Blast-resistant	*Pid3*	Li et al., 2020
*indica*	Blast-resistant	*Pid2*	Li et al., 2020
*indica*	Grain shape	*GS3*	Li et al., 2020; Gong and Han, 2022
*indica*	Rice quality	*Chalk5*	Li et al., 2020
*indica*	P uptake	*Pup1*	Li et al., 2020
*indica*	Tolerance to nitrogen deficiency	*TOND1*	Li et al., 2020
*japonica*	Resistance to abiotic stress	*Str19*	Jing et al., 2023
*temperate japonica*	Cold tolerance	*HAN1*	Mao et al., 2019
*temperate japonica*	Cold tolerance	*OsMAPK3 and OsLEA9*	Lou et al., 2022
*temperate japonica*	Cold tolerance	*Ctb1, CTB2, CTB4a, bZIP73, LTT1*	Lou et al., 2022
*temperate japonica*	Dense and erect panicle	*DEP1*	Huang et al., 2009
*temperate japonica*	Tiller angle	*TAC1*	Yu et al., 2007
*temperate japonica*	Plant architecture	*IPA1*	Jiao et al., 2010
*temperate japonica*	Gelatinization temperature	*ALK*	Gao et al., 2003
*temperate japonica*	Grain weight	*GW8*	Li et al., 2020
*temperate japonica*	P uptake	*Phr1*	Li et al., 2020
*temperate japonica*	Cold tolerance	*COLD1*	Li et al., 2020
*temperate japonica*	Narrow leaf, grain number, Grain yield	*NAL1*	Li et al., 2020
*temperate japonica*	Grain weight	*GW5*	Li et al., 2020
*temperate japonica*	Cold tolerance	*CTB4a*	Li et al., 2020
*tropical japonica*	Drought-tolerant	*qDTY3.2*	Acuña et al., 2008; Grondin et al., 2018
*tropical japonica*	Blast-resistant	*chitinase, HSP90, OXO, GLP, PR5, POX*	Carrillo et al., 2021
*tropical japonica*	Black pericarp	*Kala4*	Oikawa et al., 2015

Apart from the heterosis observed in hybrids resulting from the genetic differentiation between two *indica* heterotic groups: short-statured varieties of South China origin and medium-height lines of Southeast Asia origin (Xie et al., 2015), inter-subspecific hybrids between *indica* and *japonica* rice varieties exhibited significantly superior performance and higher heterosis compared to intra-subspecific hybrids (Ouyang et al., 2022). With the gradual resolution of hybrid sterility issues (Zhang, 2020; Ouyang et al., 2022; Wang et al., 2023b), the utilization of heterosis from inter-subspecific hybrids between *indica* and *japonica* rice has emerged as a feasible strategy for developing hybrid rice with higher yield potential. In South China, a series of *indica*-*japonica* inter-subspecific hybrid rice varieties, exampled by Yongyou series of intersubspecific hybrid rice, have been bred through introgression and fixation of advantageous alleles related to hybrid sterility and key yield-determining genes into both male and female parents (Wang et al., 2022a). The effective pyramiding of rare superior alleles with positive dominance effects in hybrids has contributed to higher yields. With the discovery of favorable rare alleles in both *indica* and *japonica* rice varieties, it is anticipated that *indica-japonica* hybrid rice will exhibit even better performance in the future (Wei and Huang, 2019).

Genomic investigation has provided evidence of apparent introgression of heterotic loci from other subgroups into *indica* hybrid rice parents (Lin et al., 2020), suggesting the presence of potential heterotic loci among subgroups and indicating that hybrid vigor among the six subgroups could be expected.

## 6 The knowledge challenge to understand introgression among subgroups and its limitation

Genomic advances have provided rich information on genetic diversity and subgroup divergence within the Asian cultivated rice, leading to the proposal of more subgroups beyond the two subspecies, *indica* and *japonica*. This provides valuable guidance for exploiting favorable variants for further genetic improvement. Although the six subgroups, including the ‘mini’ groups, *aus*, *basmatic*, and *rayada*, exhibit distinguishable genealogies, genomic information for these subgroups, except for the widely recognized *indica* and *japonica* rice, has only been obtained from a limited number of samples from the ‘mini’ subgroups. This may not be sufficient to fully reflect and represent the genetic characteristics and diversity of these subgroups.

Although genomic evidence provides suggestions on genetic divergence in the Asian cultivated rice, it could be further substantiated by additional evidences, such as reproductive barriers, which are important indicators of genetic divergence and speciation in plant evolution. Hybrid sterility is the phenomenon where the F_1_ plant produces partially abortive male or female gametes, while its parents possess normal gametes (Kato et al., 1928). It represents a common reproductive barrier among distant varieties and poses a major obstacle to harnessing hybrid vigor between *indica* and *japonica* rice. Severe hybrid sterility typically leads to spikelet sterility in F_1_ plants derived from crosses between *indica* and *japonica* rice. Therefore, studying the phenomenon of hybrid sterility is not only beneficial for understanding the degree of genetic differentiation among subgroups of Asian cultivated rice, but also crucial for overcoming hybrid sterility and achieving the utilization of their heterosis. Twenty-four hybrid sterility loci have been identified between *indica* and *temperate japonica*, representing the highest number of such loci among the subgroups compared. Eight loci were found between *temperate japonica* and *tropical japonica*, two between *tropical japonica* and *aus*, two between *indica* and *aus*, five between *temperate japonica* and *aus*, and four between *tropical japonica* and *indica*. Strikingly, three loci were also discovered within the *indica* subgroup, suggesting potential genetic divergence within this subgroup (Zhang et al., 2022). Although genomic evidence has suggested that mini-subgroups such as *aus*, *basmatic*, and *rayada* possess unique genetic lineages (Jing et al., 2023), little work has focused on hybrid sterility in these mini-subgroups. Consequently, only a few hybrid sterility loci related to *aus* and *tropical japonica* have been detected. To date, there are limited reports (Wang et al., 2023c; Lv et al., 2024) on hybrid sterility associated with *basmatic*, and even fewer on the rarely studied subgroup, *rayada*.

Besides hybrid sterility, various other reproductive barriers have been observed in rice, including hybrid weakness (Oka, 1957; Sato and Morishima, 1987), hybrid breakdown (Oka, 1957; Oka and Doida, 1962; Sato and Morishima, 1987; Fukuoka et al., 1998; Kubo and Yoshimura, 2002), transmission ratio distortion (Nakagahra, 1972), certation (Song et al., 2002), and reciprocal gene loss of duplicated genes (Mizuta et al., 2010). Genes responsible for reproductive barrier traits are termed “speciation genes,” which contribute to the cessation of gene flow between populations and can offer clues regarding the ecological settings, evolutionary forces, and molecular mechanisms that drive the divergence of populations and species (Rieseberg and Blackman, 2010).

Past efforts in utilizing rice heterosis have primarily concentrated on intra-subspecific *indica*-*indica* hybrid vigor, primarily attributed to rarely hybrid sterility among varieties within it. Advancement on inter-subspecific *indica*- *temperate japonica* hybrid vigor in China (Wang et al., 2022a) provided a way for effectively overcoming hybrid sterility, and harnessing heterosis among sub-groups in the Asian cultivated rice. However, little attention has been paid to heterosis among all six subgroups, due to a lack of comprehensive understanding of genetic differentiation and reproductive barriers among these divergent subgroups.

Therefore, future efforts should be focused on the following: 1) identifying more unique advantageous traits/alleles within each subgroup; 2) analyzing whether there exists hybrid vigor/heterotic loci among the six subgroups; 3) studying the genetic nature of reproductive barriers, particularly hybrid sterility, among subgroups in order to deeply understand genetic differentiation, overcome reproductive barriers, achieve effective introgression of target genetic variations, and ultimately utilize the hybridization vitality among subgroups or subpopulations.

## 7 Conclusion

Crop diversity underpins the productivity, resilience, and adaptive capacity of agricultural systems. Genetic diversity, on the other hand, represents a dynamic process in crop evolution. Plants can be considered as evolving along two dimensions: anagenesis and cladogenesis. Integrating favorable traits from both evolutionary dimensions to reconstruct new combination blocks for genetic improvement is a major and effective approach in rice breeding, and this method is proving successful in *indica*-*japonica* introgression breeding practices, especially exampled in breeding programs in Northeast China. Artificial introgression accelerated evolution. Genomic advances have enhanced our understanding of the greater genetic divergences and diversity among subgroups of the Asian cultivated rice, suggesting that there is further potential for genetic variation awaiting utilization in future breeding practices. This will benefit the targeted and effective exploitation of favorable variants within the germplasm of a specific subgroup. For the mini subgroups—*aus*, *basmatic*, and *rayada*—more samples should be used to further investigate and obtain more genomic and phenotypic information about their genetic differentiation, as well as to exploit favorable variants deposited within them. Research into whether there is hybrid vigor among subgroups; as well as into reproductive barrier traits, primarily manifested as hybrid sterility, will help us better understand subgroup differentiation. It will also aid in overcoming reproductive barriers to achieve effective genetic exchange among subgroups, and even harness hybrid vigor among them. Introgression breeding is also a rational approach to improving the mini subgroups for agricultural purposes.
